# Activation of microRNA-494-targeting Bmi1 and ADAM10 by silibinin ablates cancer stemness and predicts favourable prognostic value in head and neck squamous cell carcinomas

**DOI:** 10.18632/oncotarget.4365

**Published:** 2015-06-08

**Authors:** Yu-Chao Chang, Chia-Ing Jan, Chih-Yu Peng, Yu-Chi Lai, Fang-Wei Hu, Cheng-Chia Yu

**Affiliations:** ^1^ School of Dentistry, Chung Shan Medical University, Taichung, Taiwan; ^2^ Department of Dentistry, Chung Shan Medical University Hospital, Taichung, Taiwan; ^3^ Institute of Oral Sciences, Chung Shan Medical University, Taichung, Taiwan; ^4^ Oral Medicine Research Center, Chung Shan Medical University, Taichung, Taiwan; ^5^ Department of Pathology, China Medical University Hospital, Taichung, Taiwan; ^6^ Department of Pathology, China Medical University Beigang Hospital, Yunlin, Taiwan

**Keywords:** head and neck squamous cell carcinomas, tumor initiating cells, silibinin, microRNA-494

## Abstract

Tumor initiating cells (TICs) possessing cancer stemness were shown to be enriched after therapy, resulting in the relapse and metastasis of head and neck squamous cell carcinomas (HNC). An effective therapeutic approach suppressing the HNC-TICs would be a potential method to improve the treatments for HNC. We observed that the treatment of silibinin (SB) dose dependently down-regulated the ALDH1 activity, CD133 positivity, stemness signatures expression, self-renewal property, and chemoresistance in ALDH1+CD44+ HNC-TICs. Using miRNA-microarray and mechanistic studies, SB increased the expression of microRNA-494 (miR-494) and both Bmi1 and ADAM10 were identified as the novel targets of miR-494. Moreover, overexpression of miR-494 results in a reduction in cancer stemness. However, knockdown of miR-494 in CD44^−^ALDH1^−^non-HNC-TICs enhanced cancer stemness and oncogenicity, while co-knockdown of Bmi1 and ADAM10 effectively reversed these phenomena. Mice model showed that SB treatment by oral gavage to xenograft tumors reduced tumor growth and prolonged the survival time of tumor-bearing mice by activation of miR-494-inhibiting Bmi1/ADAM10 expression. Survival analysis indicated that a miR494^high^Bmi1^low^ADAM10^low^ phenotype predicted a favourable clinical outcome. We conclude that the inhibition of tumor aggressiveness in HNC-TICs by SB was mediated by up-regulation miR-494, suggesting that SB would be a valuable anti-cancer drug for treatment of HNC.

## INTRODUCTION

Head and neck squamous cell carcinomas (HNC) is the sixth most common cancer type worldwide with poor prognosis [1]. Current therapeutic methodology of HNC patients are usually consisted of extensive surgery, radiotherapy, chemotherapy, or concurrent chemo/radiotherapy [2, 3]. Most patients, however, relapse within months after radiochemotherapy [2, 3]. Despite the improvements in the diagnosis and management of HNC, long-term survival rate remains poor and more than 50% of patients die of this disease or related complications within 5 years [2, 3]. Mounting evidence has showed that the resistance to chemoradiation therapy and other currently available targeted therapies are, in part, due to the survival of a subpopulation of cells, called cancer stem cells (CSCs) or tumor initiating cells (TICs), within the heterogeneous tumor mass. TICs possesses cancer stemness and further enriches after therapy, resulting in the relapse, metastasis, and therapeutic resistance of tumors [4-6]. HNC cells that express CD44 and ALDH1, as well as are able to form spheres in suspension culture, are proposed to be used for enriching TICs in HNC (HNC-TICs) [7-10]. More importantly, HNC-TICs present elevated epithelial-mesenchymal transition (EMT) traits and are highly metastatic, tumorigenic, and resistant to radio and chemotherapies [7]. Identification of selective anti-TICs strategies would be a therapeutic intervention to improve the treatments for HNC-related malignancies.

Silibinin (SB), a polyphenolic flavonoid isolated from the milk thistle plant Silybum marianum, has been used since ancient times in traditional European medicine, and is well-known for its hepatoprotective and pleiotropic anti-carcinogenic effect on a variety of experimental cancer models including head and neck cancers [11-13]. SB exerted an inhibitory effect on invasion ability of SCC4 head and neck cancer cells by reducing phosphorylation of ERK1/2, MMP-2, and u-PA expression [11]. Substantial evidence has demonstrated that SB inhibited proliferation, migration, invasion, angiogenesis, and metastasis and induced apoptosis via modulating multiple signaling pathfways, such as notch [14], Wnt/β-catenin [15], and Stat3 [16], in cancer cells. Besides, the anti-tumorigenecity effect of SB was recently extended to TICs in colon cancer and bladder cancer [17, 18]. SB is able to inhibit cancer stemness and epithelial–mesenchymal transition in bladder cancer cells by inactivation β-catenin/ZEB1 signaling [17]. SB has been shown as potent inhibitor of colon CSCs [18]. Though substantial evidence has demonstrated that SB inhibited the cell proliferation, migration, and invasion and induced apoptosis in HNC [11], the efficacy of SB in the specific subset of HNC-TIC is still remained elusive.

MicroRNAs (miRNAs), a class of small noncoding RNAs regulating the gene expression by binding to the 3′ untranslated region (UTR) of target mRNAs, has been reported as pathogenesis signature in various types of cancers [19-21]. MicroRNA-494 (miR-494), located in human chromosome 14q32, function as tumor suppressive or oncogenic miRNAs in different types of tumors [22]. Low expression of miR-494 has been reported in prostate cancer [23], lung cancer [22], gastrointestinal stromal tumors [24], and cholangiocarcinoma [25]. In opposite, miR-494 in up-regulated in colorectal cancer [26]. Increasing reports have shown the involvements of miRNAs the regulation of TICs properties [27-29]. For example, miR200a reduced the stem-like state and epithelial-mesenchymal transition through inhibiting ZEB2 and β-catenin signalings in nasopharygeal carnicoma cells [30]. miR34a directly targets the 3′ UTR regions of CD44 and repressed cancer stemness and metastasis in prostate cancer [31]. We previously showed that suppression of miR145 enables HNCs cells to acquire TICs properties [9]. However, it is unclear whether miR-494 is involved in regulating cancer stemness.

In this report, we evaluate the efficacy of silibinin in HNC-TICs in terms of cell proliferation, TIC markers expression, self-renewal, and *in vivo* tumorigenecity. miRNAs microarray analysis of the silibinin-treated HNC-TICs revealed that miR-494 might be a novel miRNA that suppresses the TICs effect of SB in HNC-TICs. We identified Bmi1 and ADAM10 as novel direct targets of miR-494, through which miR-494 mediates silibinin-dependent inhibition of HNC-TICs. We showed that silibinin enhanced the sensitivity of HNC-TICs to chemotherapeutic. Meanwhile, suppression of miR-494 is correlated with poor patient survival and high lymph node metastatic incidence. We demonstrate the chemopreventive and chemotherapeutic effect, as well as the downstream mechanisms, of silibinin in tackling HNC-TICs *in vitro* and *in vivo*.

## RESULTS

### Silibinin preferentially eliminates cancer stemness in HNC-TICs

**Figure 1:**
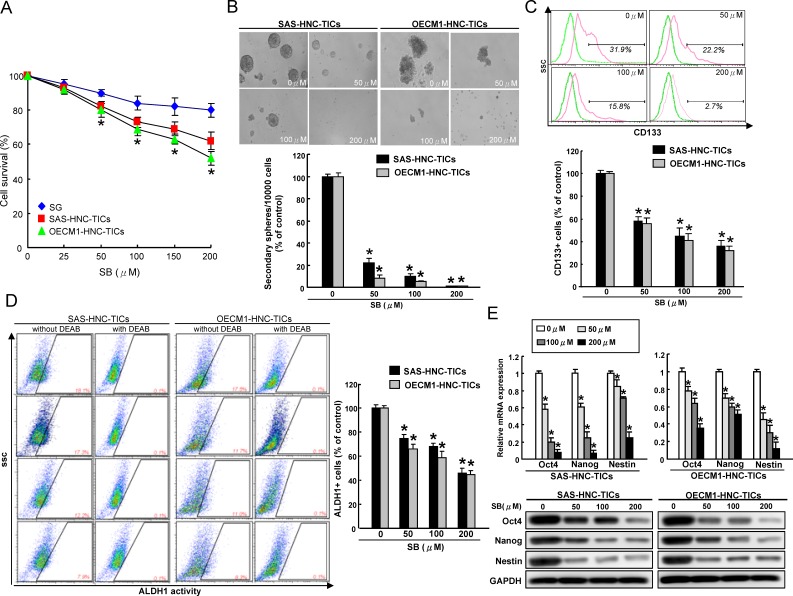
SB treatment suppresses the stem-like properties of ALDH^+^CD44^+^ HNC-TICs **A.** SG and ALDH^+^CD44^+^ HNC-TICs were treated with various concentrations of SB up to 200 μM for 24 hours. Cell survival was assessed by MTT assay and was presented as percent survival relative to untreated cells. **B.** ALDH1^+^CD44^+^ HNC-TICs treated with or without SB were subjected to a self-renewal secondary sphere-forming assay. The number of secondary spheres was calculated and was presented. The CD133 positivity **C.** and ALDH1 activity **D.** of HNC-TICs treated with or without SB was assessed by flow cytometry. **E.** The indicated stemness markers expression levels (Oct4, Nanog, and Nestin) in the SB-treated ALDH^+^CD44^+^ HNC-TICs were analyzed by quantitative real-time PCR *(upper panel*) and western blotting (*lower panel*). The experiments were repeated three times and representative results were shown. Results are means ± SD. *, *p* < 0.05 *vs*. Control.

ALDH1 and CD44 has been demonstrated to be a marker of distinguishing malignant from premalignant cells as well as identifying the putative HNC-TICs [32, 33]. We first determined whether silibinin (SB) has any cytotoxic effect to normal human oral epithelial cells (SG) and HNC cells-isolated ALDH1+ CD44+ HNC-TICs. As shown in Figure 1A, SB could suppress cell proliferation of two ALDH1+CD44+ HNC-TICs (SAS and OECM1) in a dose-dependent manner by MTT assay (Figure 1A). The effect of SB on normal human oral epithelial cells (SG) revealed that this compound did not have any significant cytotoxicity on these cells (Figure 1A). SB also displayed cytotoxic effects to parental HNC cell lines (SAS and OECM1) (Suppl. Figure 1A). Successful sphere formation phenotypes after serial passages of culture is one of indexes for evaluating the persistent self-renewal capacity of TICs [34]. To investigate the effect of SB in repressing self-renewal of HNC-TICs, we evaluated the secondary sphere-forming ability with SB treatment in HNC-TICs. In HNC-TICs dose-dependently treated with SB, the sphere-forming ability among the HNC-TICs was consistently impaired (Figure 1B). The HNC cells with TIC properties have been also isolated by sorting the cells expressing specific marker, CD133 [35]. Our data suggested SB treatment significantly decrease CD133 (Figure 1C) and ALDH1 (Figure 1D) activity of both HNC-TICs in a concentration-dependent manner. To further determine whether the reduction in TICs efficiency with SB treatment due to decreased stemness markers expression, stemness genes (Oct-4, Nanog, and Nestin) of HNC-TICs with control (DMSO) and different concentration of SB treatment were determined by real-time PCR and western blot analysis. The results confirmed that SB-treated HNC-TICs markedly reduced the mRNA and protein expression level of Oct-4, Nanog, and Nestin in both HNC-TICs (Figure 1E). In summary, down-regulation of cancer stemness by SB in HNC-TICs suggests SB treatment as potential compound on eliminating TICs in HNC.

### Anti-invasive and reversal EMT activity of SB in HNC-TICs

**Figure 2:**
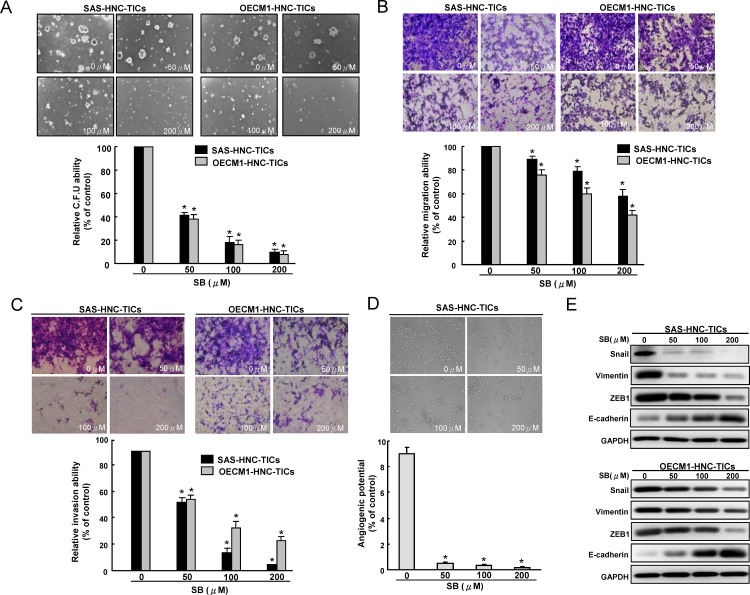
Oncogenicity and EMT traits of HNC-TICs are abolished by SB treatment Different concentration SB-treated HNC-TICs were subjected to soft agar colony formation assay **A.**, migration assay **B.**, matrix invasion assay **C.**, and vasculogenic mimicry assay **D.**. **E.** immunoblotting analysis of EMT-related markers (Snail, ZEB1, Vimentin, and E-cadherin) in control and SB-treated HNC-TICs was determined. The experiments were repeated three times and representative results were shown. Results are means ± SD. *, p < 0.05 vs. Control.

Since TICs appear to play a significant role in tumorigenesis and metastasis [36], we sought to measure the effects of SB on *in vitro* oncogenicity of HNC-TICs. Overall, our data indicate that SB dose-dependently inhibits tumor-initiating activity including colony formation (Figure 2A) and migration (Figure 2B) and invasion (Figure 2C) abilities of HNC-TICs. Recent studies indicated that glioma or ovarian TICs could differentiate into vasculogenic mimicry [37, 38]. Whether HNC-TICs contribute to vasculogenic mimicry remain unclear. HNC-TICs were able to form vessel-like structures (Figure 2D). SB treatment caused inhibition of vasculogenic mimicry of HNC-TICs (Figure 2D). Epithelial mesenchymal transition (EMT), a de-differentiation program that converts adherent epithelial cells into individual migratory cells, is thought to be a key step in the induction of cancer stemness [39, 40]. Since we have found that the effect of SB on migratory/invasion ability in HNC-TICs, we then keep on exploring whether the SB-mediated TICs depends on EMT pathway. Real-time RT-PCR analysis demonstrated down-regulation of mesenchymal-like (ZEB1, Snail, and Vimentin) transcript was seen in HNC-TICs with SB treatment (Suppl. Figure 1B). With western blotting, we demonstrated that SB treatment down-regulated a pattern of mesenchymal-like proteins (ZEB1, Snail, and Vimentin) and induced epithelial protein (E-cadherin) in HNC-TICs (Figure 2E). SB pre-treated HNC-TICs dramatically decreased tumor volume in the xenograft (Suppl. Figure 1C).

### Enhanced chemosensitivity and apopotosis in HNC-TICs by SB

**Figure 3:**
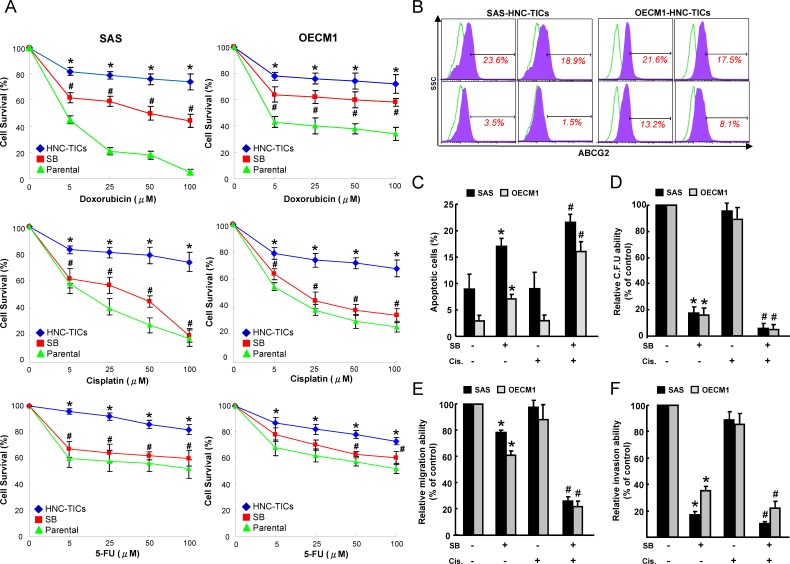
SB sensitized HNC-TICs to conventional chemo-treatment **A.** Parental HNC cells and HNC-TICs with control or SB treatment were subjected to treatment with different concentrations of doxorubicin or cisplatin or 5-FU for cell viability assessment. *, *p* < 0.05 HNC-TICs *vs*. Parental; #, *p* < 0.05 SB *vs*. HNC-TICs. **B.** The expression levels of ABCG2 in the cells indicated were determined by flow cytometry analysis. **C.** Annexin V-positive apoptosis cells were assessed in HNC-TICs synergetically treated with SB combined with cisplatin chemotherapy. Colony-forming ability **D.**, migration **E.**, and invasion ability **F.** was assessed in HNC-TICs synergetically treated with SB combined with cisplatin chemotherapy. *, *p* < 0.05 SB *vs*. HNC-TICs; #, *p* < 0.05 SB+Cisplatin *vs*. SB alone.

Recurrence of cancers after conventional therapeutic treatments is thought to be due to re-emergence of chemotherapy-resistant TICs [41]. As expected, HNC-TICs were more chemoresistant compared with the parental HNC cells. Importantly, cell viability assays showed that SB ameliorated the drug resistance of HNC-TICs to doxorubicin or cisplatin or fluorouracil (5-FU) treatment (Figure 3A). Flow cytometry analysis indicated that, in HNC-TICs treated with SB treatment, the percentage of ABCG2 positivity was reduced (Figure 3B). The combination SB and cisplatin treatment also showed a synergistic effect in promoting apoptosis in HNC-TICs (Figure 3C). Treatment with cisplatin alone did not affect the clonogenicity in HNC-TICs, the combination of SB and cisplatin co-treatment enhanced the efficacy of these treatments (Figure 3D). Meanwhile, similar synergistic effect of SB and cisplatin chemo-treatment was also observed in migration (Figure 3E) and invasion (Figure 3F) assay. Taken together, SB exhibited a prominent therapeutic effect in enhancing the sensitivity of chemotherapy in HNC-TICs.

### SB-inducible miR-494 directly targets Bmi1 and ADAM10

**Figure 4:**
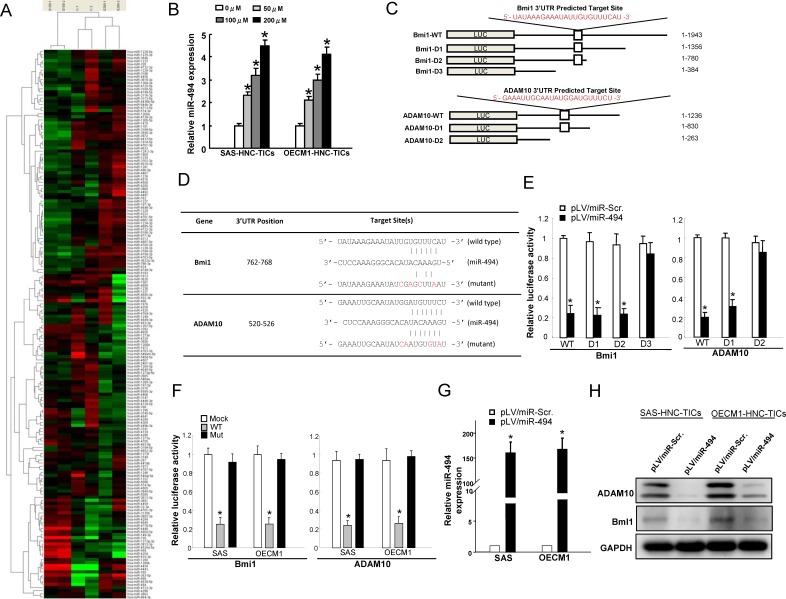
SB activates miR-494 and miR-494 direct targets Bmi1 and ADAM10 **A.** The indicated miRNAs expression levels in the SB-treated HNC-TICs were analyzed by miRNAs microarray analysis. **B.** qPCR analysis was applied to analyzed the relative miR-494 expression level in SB dose-dependently treated HNC-TICs. **C.** The wild-type (WT) and serial deleted forms of the 3′UTR reporter Bmi1 and ADAM10 plasmids were constructed as shown in the schematic presentation. **D.** Schematic presentation of the constructed Bmi1 and ADAM10 3′UTR reporter plasmids were used in this study. **E.** The wild-type and deleted forms of the Bmi1 and ADAM10 reporters were co-transfected with miR-494 or empty vector into HNC-TICs. The luciferase activity of each combination was assessed and was presented; **F.** Similar reporter assays were performed in HNC-TICs with wild-type (WT) and mutated (Mut) reporter plasmids. The results of the luciferase assays indicated that only WT reporter activity was inhibited by miR-494. **G.** Expression level of miR-494 in HNC-TICs transfected with pLV-miR-scrambled (pLV-miR-Scr.) and pLV-miR-494. (H) The protein expression levels of ADAM10 and Bmi1 in miR-494-transfected HNC-TICs were analyzed by western blot.

miRNAs correlate several aspects of cancer development such as tumor cell proliferation, self-renewal, motility, epithelial-mesenchymal transition, immune evasion, and drug-resistance, which are all defined features for cancer stemness [27, 29]. Increasing reports have shown the involvements of miRNAs in the anti-tumor effects of SB in several types of malignant cancers [42]. However, the miRNAs that mediate SB-dependent regulatory mechanisms in HNC-TICs remain unclear. Control and SB-treated HNC-TICs were subjected to miRNAs microarray analyses to attempt to identify the SB-modulated specific miRNAs that mediate cancer stemness of HNC-TICs (Figure 4A). HNC-TICs with SB treatment resulted increase in the levels of various miRNA, including miR-363-5p, miR-4443, miR-4448, miR-4454, miR-720, miR-668, and miR-494 (Figure 4A). Results showed that miR-494 expression was significantly increased in HNC-TICs with SB dose-dependent treatment (Figure 4A). Real-time RT-PCR analysis further confirmed that SB treatment showed a dose-dependent increase in the levels of miR-494 expression in HNC-TICs (Figure 4B). Using the Target Scan program, we identified potential miR-494 targeting sites in the 3′UTR regions of Bmi1 and ADAM10. Bmi-1, a member of the Polycomb (PcG) family of transcriptional repressors, mediates gene silencing by regulating chromatin structure [43]. Bmi-1 was shown being involved in tumor initiation, self-renewal, and metastasis in malignant carcinomas including HNC [44]. Strong expression of Bmi1 is shown as an indicator of a poor prognosis for HNC patients [45]. A disintegrin and metalloprotease 10 (ADAM10), membrane-bound cell surface glycoproteins, has been shown to be responsible for angiogenesis, development, angiogenesis, and tumorigenesis [46]. The expression of ADAM10 is correlated with stemness properties in normal stem cells and cancer stem cells [47, 48]. Silencing ADAM10 effectively attenuated migration and invasiveness in HNC cells through MMPs up-regulation [49]. It is unclear whether miR-494 directly targets the 3′UTR of Bmi1 and ADAM10. Further, we constructed reporter plasmids containing either full-length, serial deletion (the potential miR-494 targeting site was deleted) (Figure 4C), or mutated forms of the 3′UTR region of Bmi1 and ADAM10 (Figure 4D). Luciferase reporter assays demonstrated that miR-494 reduced the luciferase activity of reporter plasmids containing full-length Bmi1 and ADAM10 3′UTR (Figure 4E & Figure 4F). However, when the potential Bmi1 and ADAM10 targeting site was deleted or mutated, miR-494 no longer inhibited the luciferase activity (Figure 4E & Figure 4F). Consistently, the protein levels of ADAM10 and Bmi1 were decreased in the miR-494-overexpressing HNC-TICs (Figure 4H). These results identified a crucial miR-494 binding site on the 3′UTR of Bmi1 and ADAM10 to suppress its expression. Consistently, real-time RT-PCR and western blotting analysis showed that SB treatment of HNC-TICs also suppressed the mRNA and protein levels of Bmi1 and ADAM10, which our data implicated as targets of miR-494 (Suppl. Figure 1D & Suppl. Figure 1E).

### Bmi1 and ADAM10 are targets of miR-494-mediated cancer stemness

**Figure 5:**
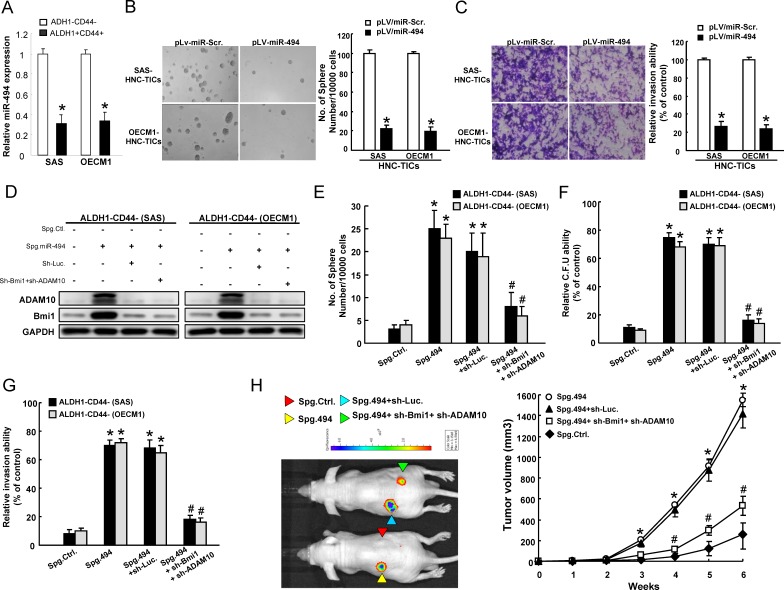
miR-494 modulates cancer stemness through targeting Bmi1 and ADAM10 **A.** miR-494 expression in ALDH^+^CD44^+^ and ALDH1^−^CD44^−^ cells was assessed by quantitative real-time PCR and presented as relative fold-changes. **B.** HNC-TICs with pLV-miR-Scr. and pLV-miR-494 were subjected to a sphere formation assay. The quantitative sphere number was presented in the chart at the right. Only spheres with a diameter more than 50 μm were counted. **C.** HNC-TICs transfected with pLV-miR-Scr. or pLV-miR-494 were then subjected to invasion assay. **D.** Spg-miR-494-ALDH1-CD44- non-HNC-TICs with or without co-knockdown of Bmi1 and ADAM10 expression were subjected to western blotting to assess the expression level of Bmi1 and ADAM10. ALDH1^−^CD44^−^ HNC cells transfected with indicated plasmids were subjected to a sphere formation assay **E.**, colony formation assay **F.**, invasion assay **G.**, and the tumor measurements in xenografts for 6 weeks **H.**. *, *p* < 0.05 Spg. 494 or Spg. 494+sh-Luc. *vs*. Spg. Ctrl.; #, *p* < 0.05 Spg. 494+sh-Bmi1+sh-ADAM10 *vs*. Spg. 494+sh-Bmi1+sh-ADAM10.

Quantitative RT-PCR analysis to confirm that miR-494 levels were low in ALDH1^+^/CD44^+^ and sphere-forming HNC-TICs but high in ALDH1^−^/CD44^−^ and parental cells (Figure 5A ; Suppl. Figure 2A). To determine functional role of miR-494 in cancer stemness, we then exogenously overexpressed miR-494 in HNC-TICs, and showed that overexpressed miR-494 decreased sphere-forming ability (Figure 5B), side population (Suppl. Figure 2B), as well as the invasion ability (Figure 5C) in HNC-TICs. The limiting dilution xenotransplanted mice assay showed that as few as 100 ALDH+CD44+ HNC-TICs formed tumors when injected into nude mice, the tumor initiation capacity in HNC-TICs was suppressed by miR-494-overexpression (Suppl. Figure 2C). In contrast, suppressing miR-494 expression with an miRNA SPONGE (Spg-miR-494) in CD44^−^ALDH1^−^ non-HNC-TICs (Suppl. Figure 2D) dramatically resulted in increased Bmi1 and ADAM10 protein levels, while co-knockdown of Bmi1 and ADAM10 reversed this effect (Figure 5D). Silencing of endogenous miR-494 induced self-renewal capability in CD44^−^ALDH1^−^ cells, which would be blocked by co-knockdown of Bmi1 and ADAM10 treatment (Figure 5E). Consistently, co-knockdown Bmi1 and ADAM10 expression decreased the enhancing effect of Spg-miR-494 on clonogenicity (Figure 5F) and invasiveness (Figure 5G) in ALDH1-CD44- cells. To further link this regulatory mechanism to *in vivo* tumorigenesis in HNC-TICs, we subcutaneously transplanted parental CD44^−^ALDH1^−^, CD44^−^ALDH1^−^/Spg-Ctrl, CD44^−^ALDH1^−^/Spg-miR-494, CD44^−^ALDH1^−^/Spg-miR-494+sh-Luc., and CD44^−^ALDH1^−^/Spg-miR-494+sh-Bmi1+sh-ADAM10 cells in immunocompromised mice and monitored the tumor growth up to 6 weeks. Spg-miR-494 enhanced the tumor growth; while sh-Bmi1+sh-ADAM10 co-knockdown inhibited the Spg-miR-494-induced tumor growth (Figure 5H). Taken together, our results demonstrated a novel miR-494-mediated cancer stemness effect through on the regulation of Bmi1 and ADAM10 expression.

### Oral-feeding SB impaired tumor growth and improved the survival of HNC-TICs tumor-bearing mice through miR-494-targeting Bmi1 and ADAM10

**Figure 6:**
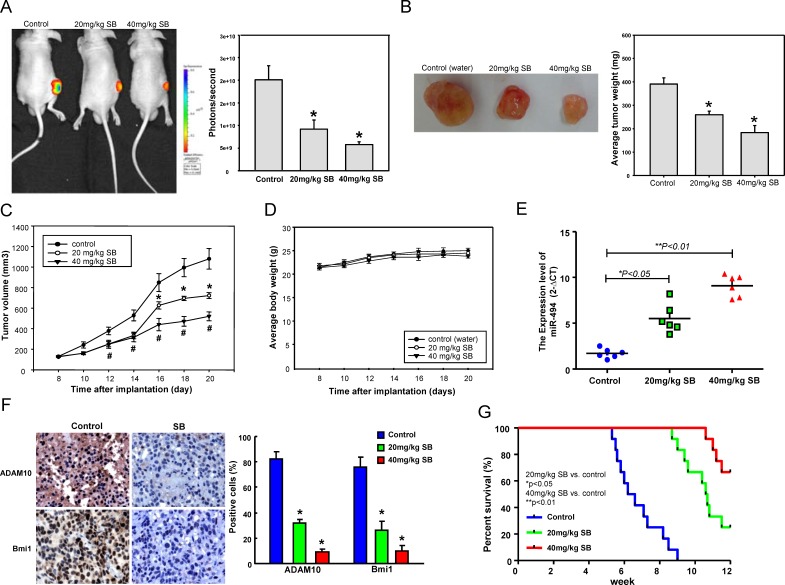
Oral delivery SB treatment suppresses tumor growth and increases animal survival After subcutaneous implantation of HNC-TICs, BALB/c nude mice (*N* = 5 for each group) were oral-feeding treated with saline or SB and then analyzed for the bioluminescence signal **A.**, average tumor weight **B.**, growth of tumor **C.**, and average mice body weight **D.**. The emitted by the implanted HNC-TICs was monitored for 20 days and was photographed. Mice were sacrificed, and tumor sections as indicated treatments were assessed for relative miR-494 expression **E.** and stained using specific antibodies against Bmi1 and ADA10 by immunohistochemistry **F.**. **G.** The survival rate of the mice treated with saline or SB was monitored for up to 12 weeks and is presented in the graph (each group; *n* = 12).

To verify the in anti-tumor effects of SB against HNC-TICs *in vivo*, immunocompromised mice bearing HNC-TIC xenografts were treated with water or SB by oral gavage. Notably, tumor formation in all recipients was reduced following xenotransplantation of HNC-TICs that received oral gavage SB treatment on day 20 as compared to control animals (Figure 6A). Moreover, by day 20, SB feeding dose-dependently induced a reduction in tumor weight (Figure 6B) and tumor volume (Figure 6C) and without any apparent signs of toxicity as evidenced by body weight monitoring (Figure 6D). Throughout the experiment, real-time RT-PCR analysis and immunohistochemical (IHC) analysis of the pathologic sections of these tumors showed that SB-treated tumor had increased miR-494 expression (Figure 6E) and decreased Bmi1 and ADAM10 expression (Figure 6F) in comparison to those from control HNC-TICs tumors. By monitoring the SB-treated mice for up to 12 weeks, we observed that administration of SB prolonged animal survival to a greater extent than did the control treatments (Figure 6G).

### An expression pattern of miR494^low^Bmi1^high^ADAM10^high^ predicts favourable prognosis

**Figure 7:**
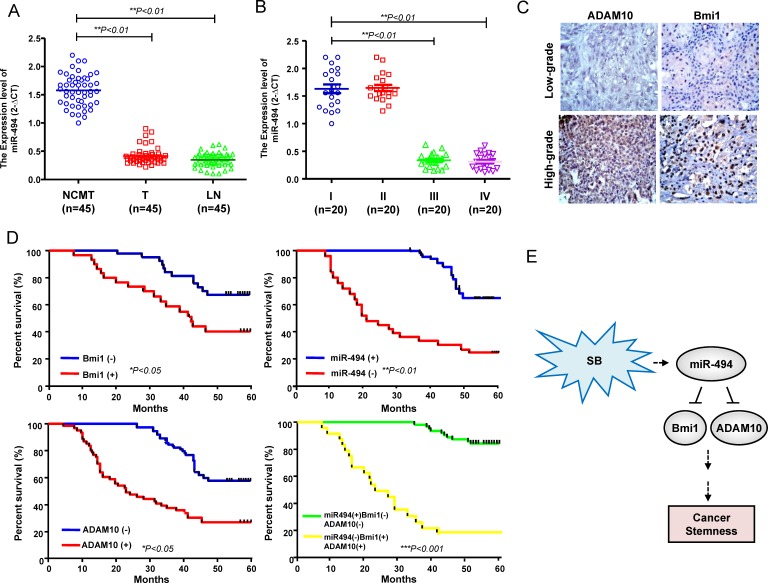
Clinical significance of miR494^low^Bmi1^high^ADAM10^high^ signature expression in specimens from HNC patients **A.** Adjacent adjacent noncancerous matched tissues (NCMT; *n* = 45), and paired tissue samples from tumor (T; *n* = 45) as well as lymph node metastatic (LN; *n* = 45) lesions in HNC patients were subjected to analysis for the expression levels of miR-494. **B.** Samples from patients with different stages (stage I to IV) of HNC were collected and subjected to qPCR for miR-494 level. **C.** IHC staining to assess the protein expression of Bmi1 and ADAM10 in high-grade and low grade HNC patient specimens. **D.** An overall survival correlation analysis was performed for HNC patient samples expressing different levels of the indicated molecules. The miR494^low^Bmi1^high^ADAM10^high^ signature correlated with the worst survival rate. **E.** A schematic representation of the SB-activated miR-494-targeting Bmi1 and ADAM10 signaling proposed in the current study.

Compared with adjacent noncancerous matched tissues (NCMT) from the same patient, the expression of miR-494 was decreased in all of the local tumor (T) samples and metastatic lymph nodes (LN) (Figure 7A). Further analysis of miR-494 expression levels in different stages of HNC specimens revealed a correlation between low miR-494 expression levels and advanced stage (Figure 7B). Immunohistochemistry analysis showed that Bmi1 and ADAM10 expression was highly expressed in high-grade HNC specimens, compared with the low-grade counter parts (Figure 7C). Kaplan-Meier survival analysis of HNC patients showed that high-expression for Bmi1, ADAM10, had a reduced survival rate; whereas patients with low expression of miR-494 had a poor survival rate (Figure 7D). Most of importance, patients with an expression profile of miR494^low^Bmi1^high^ADAM10^high^ had the lowest survival rate compared with those with miR494^high^Bmi1^low^ADAM10^low^ (Figure 7D). These findings revealed a miR494^low^Bmi1^high^ADAM10^high^ as a predictor of a poor outcome of HNC patients. Collectively, these data indicate silibinin inhibited HNC tumorigenicity through the activation miR-494-targeting Bmi1 and ADAM10 expression, which resulted in the inhibition of cancer stemness (Figure 7E).

## DISCUSSION

Aberrant microRNA-494 (miR-494) expression has been shown to regulate cancer tumorigenicity in several types of cancer through modulating target genes and multiple signaling pathways [23, 50, 51]. For example, miR-494 inhibits proliferation rate in gastrointestinal stromal tumors cells through directly repression of the KIT [24]. MiR-494 has also been demonstrated to attenuates tumor growth and metastasis *in vitro* and *in vivo* by targeting FOXM1 [52]. However, the role of miR-494 in HNC-TICs remains unclear. In this study, we showed that miR-494 directly binds to the 3′UTR regions of Bmi1 and ADAM10 in HNC-TICs (Figure 4), thus represses the tumorigenecity and TIC properties such as sphere formation capability, CD44 and ALDH1 expression, clonogenic ability, and *in vivo* tumor initiation incident (Figure 5). Co-knockdown of Bmi1 and ADAM10 rescued the induction effect of spg-miR-494 on TIC properties (Figure 5). Kaplan-Meier survival analysis showed that the overexpression of Bmi1, ADAM10, or underexpression of miR-494 had a shorter overall survival in HNC patients; miR494^low^Bmi1^high^ADAM10^high^ signature presented the worst outcome in HNC patients (Figure 7D). Meanwhile, the result showed a pattern of low miR-494 expression in the metastatic and advanced stage tissues (Figure 7A). Clinical results suggested that a miR494^low^Bmi1^high^ADAM10^high^ signature could be a potential predictor for disease progression and poor clinical outcome of HNC patients (Figure 7D). To our knowledge, this is the first report demonstrating the regulatory role of the miR494-targeting Bmi1 and ADAM10 expression in the regulation of cancer stemness (Figure 7E). This report identified a novel miR-494-dependent tumor suppression pathway that is able to eliminate the persistent TIC cells.

Mounting studies have suggested that dietary compounds interfere with tumor-initiating- and cancer stemness-related pathways, and therefore offer a promising approach for cancer prevention [34]. Silibinin (SB) has been recognized as a promising anticancer drug and is being developed as a chemopreventive agent in various cancers [11-13]. *In vitro* and *in vivo* studies showed that SB has anti-oxidant, anti-inflammatory, anti-proliferative, and pro-apoptotic activities [11-13]. Interestingly, SB also inhibits TIC-like properties in colon and bladder cancers [17, 18]. We found that greater inhibition effect on HNC-TICs in terms of survival upon SB treatment; while normal oral epithelial cells were not repressed by this compound. SB seems specifically reduced the sphere formation, colony formation, and tumor-initiation abilities in HNC-TICs (Figure 1). Increasing reports have shown the involvements of miRNAs in the anti-tumor effects of SB. SB was also reported to inhibit erlotinib-resistance in EGFR mutant non-small cell lung cancer cells by switching on suppressor miRNAs such as miR-200c [42]. In the present study, SB suppressed HNC cancer stemness via enhancing miR-494-mediated suppression of Bmi1 and ADAM10. We identified Bmi1 and ADAM10 as novel downstream targets of miR-494 (Figure 4). The detail role of ADAM10 in regulating cancer stemness in HNC-TICs may need further investigation.

Evidence in recent reports indicates the chemo-resistance is mainly caused by cancer stemness [41]. SB in combination with 5-fluorouracil and paclitaxel would effectively prevent the emergence of chemoresistance in renal carcinoma cells [13]. A combination of SB and doxorubicin demonstrated a significant growth suppressive effect in non small cell lung cancer (NSCLC) cells-xenograft compared to treatment with either agent alone [53]. In mouse xenograft model, oral delivery SB treatment lessened tumor initiating activity *in vivo*. However, oral feeding SB to mouse-bearing tumors did not completely lead to tumor regression of HN-CICs. In our *in vitro* data, we showed that SB synergized with cisplatin or 5-fluorouracil or doxorubicin to prevent the persistent survival of HNC-TICs and enhance the efficacy of these chemotherapies (Figure 3). It would be important to examine SB combine with chemoradiotherapeutic therapy treatment on suppressing HNC-TICs-bearing in the future. t is likely that through enhancing miR-494, SB suppresses the potentially emerging drug-resistance of HNC-TICs and allows cisplatin to eliminate HNC-TICs. Recent reports have shown that epithelial mesenchymal transition (EMT) traits are essential to maintain self-renewal, tumorigenicity, and metastasis of TICs. For example, Snail/Slug/Twist, important transcription factors of EMT, govern the development of TICs which responsible for cancer initiation, drug resistance and metastasis [39, 40]. We first demonstrated that SB inhibited mesenchymal markers (ZEB1, Snail, and Vimentin) expression and up-regulated epithelial marker (E-cadherin) expression levels of HNC-TICs (Figure 2E), suggesting SB as blockers of EMT. Therefore, it is interesting to ask whether miR-494 is involved in regulating chemoresistance and EMT in HNC-TICs.

Conclusively, the present report showed that the SB effectively suppressed self-renewal, tumor-initiating, and chemoreisitance properties of HNC-TICs *in vitro* and *in vivo* partially through miR-494 activation. miR-494-targeting Bmi1 and ADAM10 expression would greatly contribute to a deeper understanding of cancer stemness acquisition in HNC, and promote the development of promising therapeutics for HNC-TICs eradication. Experimental platforms that are more close to clinical operation are expected to support the therapeutic potential of SB in HNC patents treatment.

## MATERIALS AND METHODS

### Reagents and cell culture

Silibinin was purchased from Sigma Chemical Co. (St. Louis, MO) and was dissolved in DMSO (Merck, Darmstadt, Germany) as a stock solution of 100 mM. Just before use, Silibinin was further diluted in culture medium to appropriate final concentrations. The Smulow–Glickman (S-G) human gingival epithelial cell line was original from F.H. Kasten, East Tennessee State University, Quillen College of Medicine, Johnson City, TN [54]; SAS, a high-grade tumorigenic human tongue squamous cell carcinoma, was obtained from the Japanese Collection of Research Bioresources (Tokyo, Japan) [55]; Human gingival squamous carcinoma cells (OECM-1) were provided from Dr. C. L. Meng (National Defense Medical College, Taipei, Taiwan) grown in RPMI supplemented with 10% FBS. Cells were cultured at 37°C containing 5% CO2.

### Isolation of ALDH1+ CD44+ HNC-TICs

CD44-positive and ALDH1-positive cells in HNC cells using CD44 antibody (phycoerythrin conjugated, BioLegend) and the Aldefluor assay (Stem Cell Technologies), followed by fluorescence-activated cell sorting analysis (FACS). Technologies as described previously [7].

### Cell proliferation/survival determination by MTT assay

For evaluation of cell proliferation, 1×10^4^ cells/well in 0.1 % DMSO or different concentration of silibinin-containing medium and cultured at 37°C for 24hr. Cell proliferation/survival was determined by MTT (3-(4,5-dimethylthiazol-2-yl)-2,5-diphenyl tetrazolium bromide) assay. The MTT test was conducted according to previously used protocols [34].

### Tumorsphere-forming assay

Tumor cells were dissociated and cultured as tumorspheres in modified DMEM/F-12 supplemented with N2 (R&D), 10 ng/mL epidermal growth factor (EGF, Invitrogen), 10 ng/mL basic fibroblast growth factor (bFGF, Invitrogen), and penicillin/streptomycin at 103 live cells/low-attachment six-well plate (Corning Inc.,Corning, NY), and the medium was changed every other day until the tumor sphere formation was observed in about 2 weeks. For serial passage of spheroid cells, single cells will be obtained from accurtase treated spheroids and the cell density of passage will be 10000 cells/ml in the serum-free medium as described above [7].

### Quantitative real-time reverse-transcriptase (RT)-PCR

Total RNA was prepared from cells or tissues using Trizol reagent according to the manufacturer's protocol (Invitrogen). qRT–PCRs of mRNAs were reverse-transcribed using the Superscript III first-strand synthesis system for RT–PCR (Invitrogen). qRT-PCR reactions on resulting cDNAs were performed on an ABI StepOne™ Real-Time PCR Systems (Applied Biosystems) [7]. Primer sequences are listed in Supplementary Table 1.

### Western blot

The extraction of proteins from cells and western blot analysis were performed as described. Samples (15 μL) were boiled at 95°C for 5 min and separated by 10 % SDS-PAGE. The proteins were wet-transferred to Hybond-ECL nitrocellulose paper (Amersham, Arlington Heights, IL, USA). The following primary antibodies were listed in Supplementary Table 2. Immunoreactive protein bands were detected by the ECL detection system (Amersham Biosciences Co., Piscataway, NJ, USA) [7].

### miRNAs qRT–PCR anlysis

For miR-494 levels decetion, qRT–PCR was performed using TaqMan miRNA assays with specific primer sets (Applied Biosystems, Carlsbad, Calif). All reagents and protocols were from Applied Biosystems, and detection was performed using a 7900HT fast real-time PCR system [7].

### MiR-494 Sponge

Oligos for miR-494 sponge, and scramble construction were constructed using a pcDNA 6.2-GW/EmGFP-miR plasmid (Invitrogen). MicroRNA SPONGE sequence design was based on previous report [56]. Further multiple copy amplifications were done with recovery of BamH1 and XhoI digested fragments and subcloned into pcDNA 6.2-GW/EmGFP-miR plasmid [7].

### Soft agar colony forming assay

Six-well culture dish was coated with 2 ml bottom agar (Sigma-Aldrich) mixture (DMEM, 10% (v/v) FCS, 0.6% (w/v) agar). After the bottom layer was solidified, 2 ml top agar-medium mixture (DMEM, 10% (v/v) FCS, 0.3% (w/v) agar) containing 2×10^4^ cells was added, and the dishes were incubated at 37°C for 4 weeks. Plates were stained with 0.005% Crystal Violet then the colonies were counted. The number of total colonies with a diameter ≥100 μm was counted over five fields per well for a total of 15 fields in triplicate experiments [7].

### *In vitro* cell invasion analysis

The 24-well plate Transwell® system with a polycarbonate filter membrane of 8-μm pore size (Corning, United Kingdom) was employed to evaluate the invasion ability of cells. The membrane was coated with Matrigel^TM^(BD Pharmingen, NJ, USA). The cancer cell suspensions were seeded to the upper compartment of the Transwell chamber at the cell density of 1×10^5^ in 100 μl within serum-free medium. The lower chamber was filled with serum-free medium. or media with 10% serum After 24 hours of incubation, the medium was removed and the filter membrane was fixed with 4% formalin for 1 hour. Subsequently, the remaining cells of the filter membrane faced the lower chamber was stained with Hoechst 33258 (Sigma-Aldrich). The migrated cancer cells were then visualized and counted from 5 different visual areas of 100-fold magnification under an inverted microscope [7].

### Annexin V-FITC and PI staining assay

Apoptosis was quantified by flow cytometry with annexin V-FITC and PI staining. In brief, the cells were treated with SB alone or combined cisplatin treatment for 24 h. The treated cells were collected, washed twice with PBS, and subjected to annexin V and PI staining by using Vybrant Apoptosis Assay Kit 2 (Invitrogen, Carlsbad, CA) according to the manufacturer's step-by-step protocol. Recombinant annexin V conjugated to fluorophores and Alexa fluoro 488 dye provided maximum detection sensitivity. After staining, flow cytometry was performed to quantify apoptotic cells

### Imaging measurement of tumor growth in nude mice

All procedures involving animals were in accordance with the institutional animal welfare guidelines of the Chung Shan Medical University. For the nude mice xenograft model, 5-6 weeks old immuno-deficient nude mice (BALB/c nu/nu mice) weighing 18-22 g were used. The mice were housed with a regular 12 h light/12 h dark cycle and ad libitum access to standard rodent chow diet (Laboratory Rodent Diet 5001, LabDiet, St. Louis, MO) and were kept in a pathogen-free environment at the Laboratory Animal Unit. HNC-TICs (1×10^4^cells/0.2 mL/mouse) were injected subcutaneously into the right front axilla. Eight days postimplantation, the mice were randomly divided into three groups (N = 5 for each group) and fed by oral gavage with saline (control) and silibinin (20 and 40 mg/day/kg) suspended in PBS. The day of cell implantation was designated day 0. Imaging measurement was performed using an IVIS50 animal imaging system (Xenogen Corp.). The photons emitted from the target site penetrated through the mammalian tissue and could be externally detected and quantified using a sensitive light-imaging system. The image acquisition time was 1 min. The displayed images of the tumor sites were drawn around and quantified in photons per second using Living Image software (Xenogen Corp.) The volume was calculated (according to the following formula: [length × width^2^]/2), and then analyzed using Image-Pro Plus software. Body weight was assessed daily after cell injection. After 20 days, the animals were euthanized, and the primary tumors were isolated and weighed [34].

### Immunohistochemistry

Tissue samples were spotted on glass slides for immunohistochemical staining. After deparaffinization and rehydration, tissue sections were processed with antigen retrieval by 1X Trilogy diluted in H_2_O (Biogenics) with heating. The slides were immersed in 3% H_2_O_2_ for 10 minutes and washed with PBS three times. Tissue sections were blocked with serum (Vestastain Elite ABC kit, Vector Laboratories, Burlingame, CA, USA) for 30 minutes, then incubated with the primary antibody in listed in Supplementary Table 2. PBS solution at room temperature for 2 hours. Tissue slides were washed with PBS and incubated with biotin-labeled secondary antibody for 30 min, then incubated with streptavidin-horse radish peroxidase conjugates for 30 min, and washed with PBS three times. Tissue sections were then immersed with chromogen 3-3′-diaminobenzidine plus H_2_O_2_ substrate solution (Vector^®^ DBA/Ni substrate kit, SK-4100, Vector Laboratories) for 10 minutes. Hematoxylin was applied for counter-staining (Sigma Chemical Co.,) Finally, the tumor sections were mounted with a cover slide with Gurr^®^ (BDH Laboratory Supplies, UK) and examined under a microscope. Pathologists scoring the immunohistochemistry were blinded to the clinical data. The interpretation was done in five high-power views for each slide, and 100 cells per view were counted for analysis [7].

### HNC tissues acquirement and preparation

The study was approved by the institutional review board of China Medical University Hospital. Resected tissues from HNC patients, who gave informed consent for the use of their tissue, were harvested at surgery. 45 pairs of tumor (T) and adjacent noncancerous matched tissues (NCMT) parts, as well as lymph node (LN) metastatic HNC lesions were obtained from surgical procedures send to the pathology lab for frozen section diagnosis. The information regarding the different stages of HNC patients is described in listed in Supplementary Table 3. Tumor tissues were microscopically screened to have >70% of their areas occupied by tumor cells. The remaining specimens were snap frozen in liquid nitrogen and stored at −80°C for quantitative real-time reverse transcription–PCR (qRT-PCR) (Applied Biosystems, Foster City, CA, USA) [7].

### Statistical analysis

Statistical Package of Social Sciences software (version 13.0) (SPSS, Inc., Chicago, IL) was used for statistical analysis. Student's t test was used to determine statistical significance of the differences between experimental groups; p values less than 0.05 were considered statistically significant. The level of statistical significance was set at 0.05 for all tests.

## SUPPLEMENTARY MATERIAL FIGURES AND TABLES


