# Supplementary material for: Activation of microRNA-494-targeting Bmi1 and ADAM10 by silibinin ablates cancer stemness and predicts favourable prognostic value in head and neck squamous cell carcinomas
Source: Oncotarget. 2015 Jun 8;6(27):24002–16. doi: 10.18632/oncotarget.4365 (PMC4695166; doi:10.18632/oncotarget.4365)
Supplement: Supplementary file 1 [file oncotarget-06-24002-s001.pdf]

Activation of microRNA-494-targeting Bmi1 and ADAM10 by silibinin ablates cancer stemness and predicts favourable prognostic value in head and neck squamous cell carcinomas

Supplementary Material

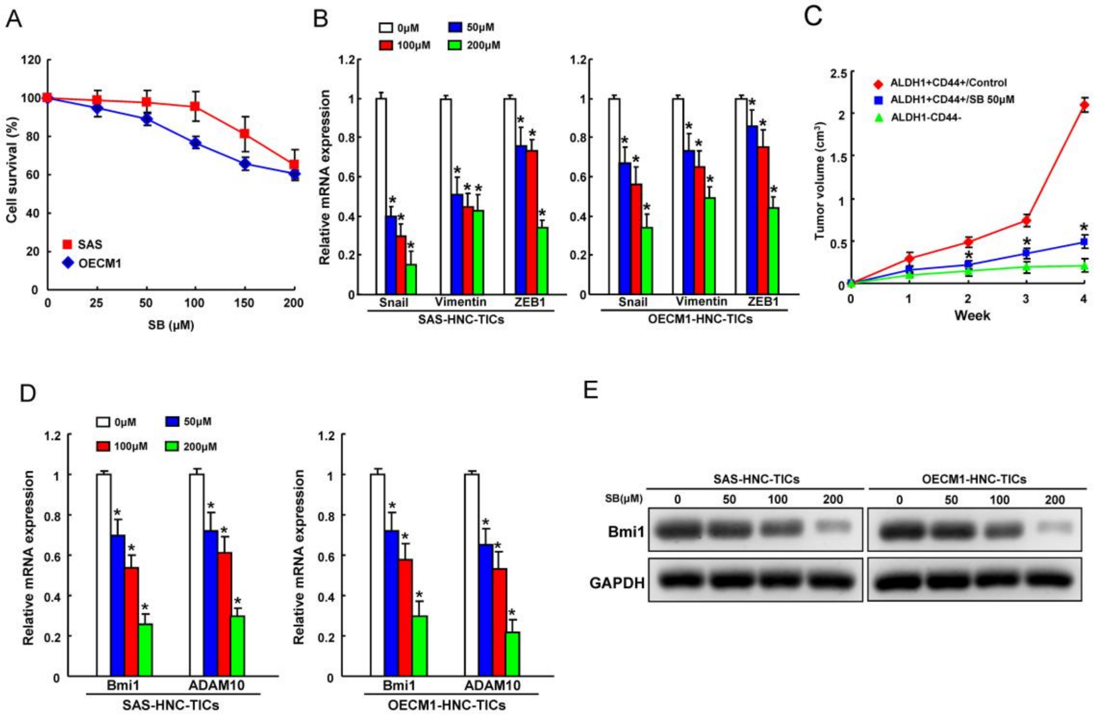

Supplemental Figure 1

(A) Cell survival of SAS and OECM1 cells treated with various concentrations of SB was assessed by MTT assay. (B) Quantitative RT-PCR of EMT-related markers (Snail, ZEB1, and Vimentin) in control and SB-treated HNC-TICs was determined. (C) SB-pretreated ALDH1+CD44+ HNC-TICs were subjected to in vivo xenograft tumor growth. Quantitative RT-PCR (D) and immunoblotting analysis (E) of Bmi1 and ADAM10 in control and SB-treated HNC-TICs was determined.

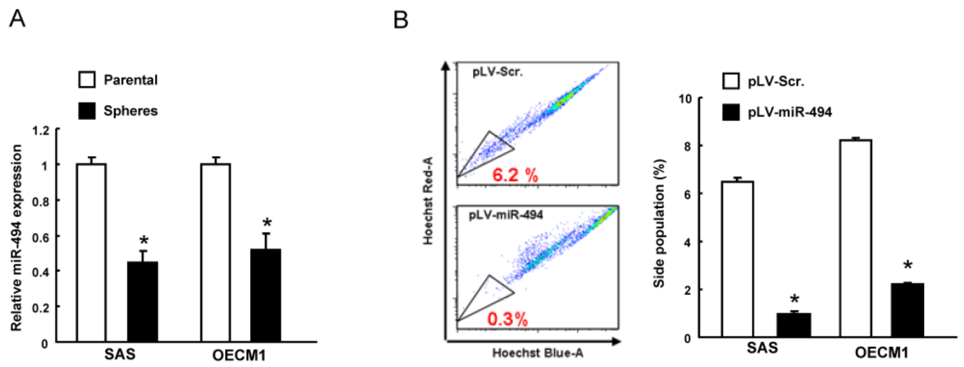

C

ALDH1+ CD44+	Injected Cells Number	pLV-miR-Scr.	pLV-miR-494
SAS	100,000	3/3	1/3
	10,000	3/3	0/3
	1,000	3/3	0/3
	100	2/3	0/3
OECM1	100,000	3/3	0/3
	10,000	3/3	0/3
	1,000	2/3	0/3
	100	1/3	0/3

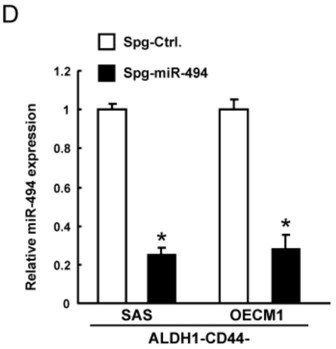

Supplemental Figure 2

(A) miR-494 expression in sphere-forming and parental cells was assessed by quantitative real-time PCR and presented as relative fold-changes. (B) Effects of miR-494 overexpression on side population. (C) Nude mice were subcutaneously injected with various numbers of pLV-Scr. and pLV-miR-494-transfected ALDH^{+}CD44^{+} HNC-TICs (from 100 to 100,000) derived from SAS and OECM1 (n=3). Mice were monitored for 4 to 12 weeks for the occurrence of tumor mass., and the tumor incidence in each group was calculated and is presented in the chart. (D) The efficiency of miR-494 repression in Spg-miR-494 ALDH1-CD44- cells was monitored by quantitative real-time PCR.

Supplementary Materials and Methods

Side population analysis

Cells were resuspended at 1×10^6/mL in pre-warmed DMEM with 2% FCS. Hoechst 33342 dye was added at a final concentration of 5μg/mL in the presence or absence of verapmil (50μM; Sigma) and was incubated at 37°C for 90 min with intermittent shaking. At the end of the incubation, the cells were washed with ice-cold HBSS with 2% FCS and centrifuged down at 4°C, and resuspended in ice-cold HBSS containing 2% FCS. Propidium iodide at a final concentration of 2 μg/mL was added to the cells to gate viable cells. The cells were filtered through a 40-μm cell strainer to obtain single cell suspension before analysis. The Hoechst 33342 dye was excited at 357 nm and its fluorescence was dual-wavelength analyzed (blue, 402–446 nm; red, 650–670 nm). Analyses were done on FACSAria (BD, San Diego, CA).

Lentiviral-mediated RNAi for silencing Bmi1 and ADAM10

The pLV-RNAi vector, which co-expressing GFP protein in infected host cells, was purchased from Biosettia Inc. (Biosettia, San Diego, CA, USA). The method of cloning the double-stranded shRNA sequence is described in the manufacturer's protocol. Oligonucleotide sequence of lentiviral vectors expressing shRNA that targets human Bmi1 and ADAM10 were synthesized and cloned into pLVRNAi to generate a lentiviral expression vector. Sh-Luc:5'-CCGGACTTACGCTGAGTACTTCGAACTCGAGTTCGAAGTACTCA GCGTAAGTTTTTTG-3' was utilized for experimental control. Lentivirus production was performed as above. Stable pLV-RNAi expressed HNSCC cell lines were further purified by cell sorting with GFP positive cells.

In vitro vasculogenic mimicry assay

The 96-well plates were coated with 50 µl of Matrigel (10 mg/ml) (BD Bioscience Pharmingen) by incubation at 37 °C for 1 h. HNC-TICs were suspended in M200 with 10% FBS and LSGS, and then plated onto a layer of Matrigel at a density of 2.5×10^4 cells/well. The plates were then incubated for 12 h at 37 °C, and capillary-like tube formation was observed under microscope.

Microarray analysis and bioinformatics

Total RNA was extracted from cells using Trizol reagent (Life Technologies, Bethesda, MD, USA) and the Qiagen RNAeasy (Qiagen, Valencia, CA, USA) column for purification. Microarray analysis was performed using the Human OneArray miRNA v2 (Phalanx Biotech, Belmont, CA, USA). Fluorescence intensities were measured and scanned separately using Molecular Dynamics Axon 4100A scanner and assessed using GenePixPro software.. Data analysis was performed using GenePix Pro 3.0.5.56 (Axon Instruments, USA) and GeneSpring GX 7.3.1 software (Agilent, Palo Alto, CA). The average-linkage distance was used to assess the similarity between two groups of gene expression profiles as described below. The difference in distance between two groups of sample expression profiles to a third was assessed by comparing the corresponding average linkage distances (the mean of all pair-wise distances (linkages) between members of the two groups concerned). The error of such a comparison was estimated by combining the standard errors (the standard deviation of pair-wise linkages divided by the square root of the number of linkages) of the average-linkage distances involved. Classical multidimensional scaling (MDS) was performed using the standard function of the R program to provide a visual impression of how the various sample groups are related.

SUPPLEMENTAL TABLES

Suppl. Table 1. The sequences of the primers for quantitative RT-PCR

Gene (Accession No.)	Primer Sequence (5' to 3')	Product size (bp)	Tm (°C)
Oct4 (NM_002701)	F: GTGGAGAGCAACTCCGATG<br>R: TGCTCCAGCTTCTCCTTCTC	86	60
Nanog (NM_024865)	F: ATTCAGGACAGCCCTGATTCTTC<br>R: TTTTTGCGACACTCTTCTCTGC	76	60
Nestin (NM_006617)	F: AGGAGGAGTTGGGTTCTG<br>R: GGAGTGGAGTCTGGAAGG	112	50
Snail (NM_005985)	F: GCTGCCAATGCTCATCTGGGACTCT<br>R: TTGAAGGGCTTTCGAGCCTGGAGAT	300	55
ZEB1 (NM_001128128)	F: TGCACTGAGTGTGGAAAAGC<br>R: TGGTGATGCTGAAAGAGACG	237	60
Vimentin (NM_003380)	F: GCAATCTTTCAGACAGGATGTTGAC<br>R: GATTTCCTCTTCGTGGAGTTTCTTC	118	55
GAPDH (NM_002046)	F: CATCATCCCTGCCTCTACTG<br>R: GCCTGCTTCACCACCTTC	180	60

Suppl. Table 2.

Protein	Assay	Antibody	Origin
Oct4	WB, IHC	rpab	NBP1-45751, Novus Biologicals, Inc
Nanog	WB	rpab	#3580, Cell Signaling Technology, Inc.
Nestin	WB	mmab	#MAB5326, Millipore, Inc.
Snail	WB	mmab	#3895, Cell Signaling Technology, Inc.
Vimentin	WB	mmab	#3390, Cell Signaling Technology, Inc.
ADAM10	WB IHC	rpab	#14194, Cell Signaling Technology, Inc.
Bmi1	WB, IHC	rpab	#6964, Cell Signaling Technology, Inc. ;
GAPDH	WB	rpab	Ab9385, Abcam, Inc

Abbreviations: WB, Western blot; mmab, mouse monoclonal antibody; rpab, rabbit polyclonal antibody

Suppl. Table 3. Clinicopathological parameters of HNC patients

Parameters	Stage I and Stage II	Stage III and IV
Age (years)	31-62	31-72
Mean age ± SD	47.6 ± 10.0	52.5 ± 10.4
Sex (M/F)	39/1	40/0
Site		
Buccal mucosa	17	16
Tongue	22	24
Other sites	1	0
Histopathologic diagnosis		
Well differentiation cancer	21	4
Moderate differentiation cancer	12	14
Poor differentiation cancer	7	22
